# Aptamer-based CRISPR-Cas powered diagnostics of diverse biomarkers and small molecule targets

**DOI:** 10.1186/s13765-023-00771-9

**Published:** 2023-02-18

**Authors:** Ulhas Sopanrao Kadam, Yuhan Cho, Tae Yoon Park, Jong Chan Hong

**Affiliations:** 1 Division of Life Science and Division of Applied Life Science (BK21 Four), Plant Molecular Biology and Biotechnology Research Center, Gyeongsang National University, Jinju, Gyeongnam-do 52828 Republic of Korea; 2 Graduate School of Education, Yonsei University, Seoul, 03722 Republic of Korea; 3 Division of Plant Sciences, University of Missouri, Columbia, MO 65211 USA

**Keywords:** Biomarker, Pathogen, Disease diagnostics, CRISPR-Cas, Cas12a, Cas13a, Cas14a, Aptamer, Fluorescence, Colorimetric detection

## Abstract

CRISPR-Cas systems have been widely used in genome editing and transcriptional regulation. Recently, CRISPR-Cas effectors are adopted for biosensor construction due to its adjustable properties, such as simplicity of design, easy operation, collateral cleavage activity, and high biocompatibility. Aptamers’ excellent sensitivity, specificity, in vitro synthesis, base-pairing, labeling, modification, and programmability has made them an attractive molecular recognition element for inclusion in CRISPR-Cas systems. Here, we review current advances in aptamer-based CRISPR-Cas sensors. We briefly discuss aptamers and the knowledge of Cas effector proteins, crRNA, reporter probes, analytes, and applications of target-specific aptamers. Next, we provide fabrication strategies, molecular binding, and detection using fluorescence, electrochemical, colorimetric, nanomaterials, Rayleigh, and Raman scattering. The application of CRISPR-Cas systems in aptamer-based sensing of a wide range of biomarkers (disease and pathogens) and toxic contaminants is growing. This review provides an update and offers novel insights into developing CRISPR-Cas-based sensors using ssDNA aptamers with high efficiency and specificity for point-of-care setting diagnostics.

## Introduction

In recent years, novel diagnostic tools empowered by the integration of CRISPR-Cas proteins (clustered regularly interspaced short palindromic repeats-CRISPR associated) have fueled several applications for food sensing and biosafety analysis [1]. CRISPR-Cas is part of the adaptive immune system of the bacteria and archaea, which protects the host from invading genetic materials, like bacteriophages or plasmids [2]. In principle, the CRISPR-associated proteins (Cas protein) use specific sequences that make up the guide RNA (gRNA) to cleave recognition sites of the foreign DNA under the control of gRNA. This effectively silences the exogenously introduced genetic elements and protects the host organism.

**Fig. 1:**
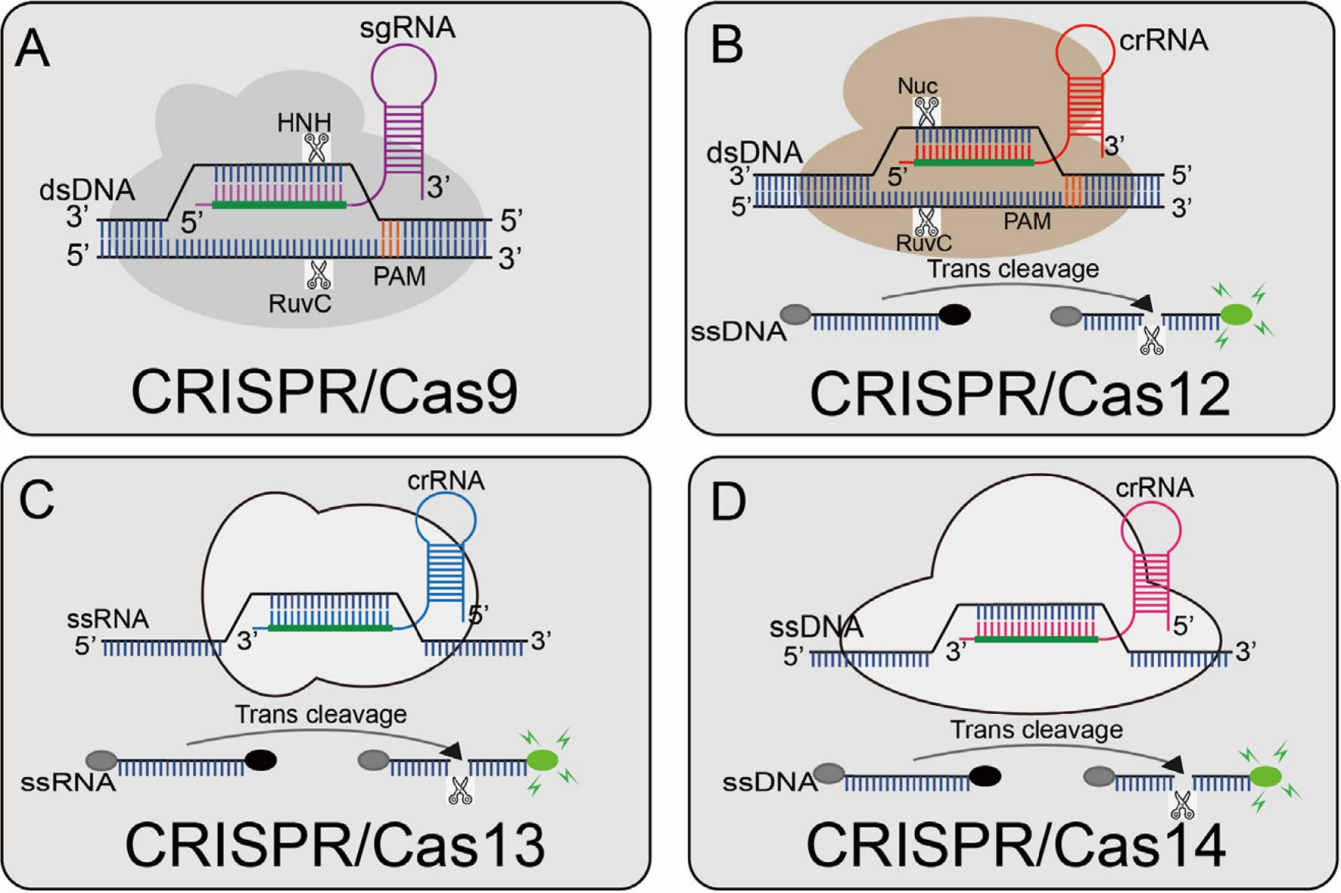
**Overview of CRISPR-Cas enzyme activities and their catalytic mechanisms.** **A** Cas9 can cleave the target and non-target strands of DNA; a short trinucleotide PAM is also essential for the initial DNA binding; **B** Cas12a can cleave dsDNA under the guidance of gRNA. The Cas12a enzyme recognizes the PAM of the original T-rich spacer and then recognizes the target sequence to generate PAM distal dsDNA breaks with staggered 5′ and 3′ ends, and Cas12 has the side chains trans-cleavage activity. At the time that the sgRNA-guided DNA is combined in Cas12, Cas12 will release a powerful, indiscriminate single-stranded DNA (ssDNA) cleavage activity; **C** Cas13 can activate its single-stranded RNA (ssRNA) cleavage activity by binding to crRNA, and it has a additional cleavage activity triggered by the target RNA; **D** Cas14 protein is a RNA-guided nuclease and can recognize the target ssDNA without restriction sequences and cleave it, and also can non-specifically cleave the surrounding ssDNA nucleases molecule (Modified after: Li et al., Diagnostics 2022, 12(10); Copyright: CC BY License)

Further, advancements in the revolutionary CRISPR-Cas-based gene editing system won it a Nobel Prize in Chemistry in 2020. This biotechnological tool has been widely adopted in genomic editing for insertion, knockout, fusion, gene regulation, epigenetic modification, targeted mutagenesis, localization, and crop improvement. Several CRISPR-Cas systems have been shown to have specific (cis-cleavage) or nonspecific (trans-cleavage or collateral-damage) degrading activity on dsDNA, ssDNA, or ssRNA. The discovery of the unusual spread of repetitive DNA elements in bacteria led to concurrent series of revelations regarding the multifunctional role of CRISPR-Cas proteins [1]. Later findings on CRISPR-Cas9 fueled the race to understand and develop CRISPR-Cas technology in gene editing under the guidance of gRNA. Subsequently, outstanding application of the CRISPR-Cas9 system for genome editing was evidenced [3], which further catapulted elaborate studies and novel applications in microbiology, plant biology, and biomedical sciences, specifically genomic editing and molecular diagnostics [4]. The RNA-guided and specific-targeted CRISPR effectors like Cas9, Cas12, Cas13, and Cas14 (Fig. 1) were successively discovered [2, 5].

In cis-cleavage, Cas proteins (CRISPR-Cas9) first recognizes the protospacer-adjacent motif (PAM) in specific dsDNA and then uses guide CRISPR-RNA (crRNA) to create a double-stranded break. Whereas, the trans-cleavage activity (collateral damage) occurs when a ternary complex of Cas, crRNA, and target nucleic acid (ssDNA or ssRNA) is formed, which then activates indiscriminate nonspecific cleavage of nearby nucleic acids (DNA or RNA). This indiscriminate nucleic acid degradation potential is coupled with fluorescence labeling of DNA probes as reporter molecules and for signal amplification (Table 1).

**Fig. 2:**
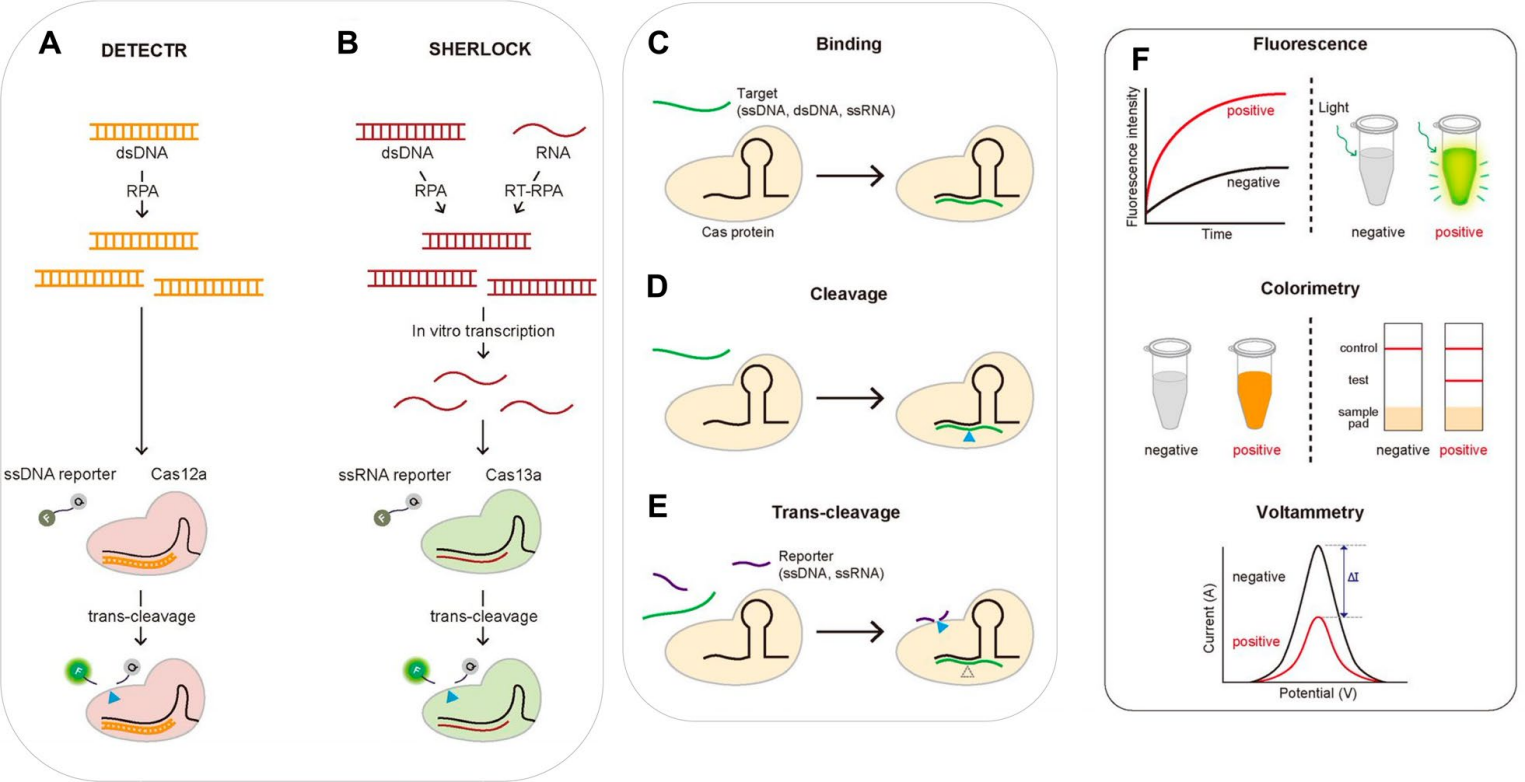
**CRISPR-based diagnostics. A**, **B** Schematic of DETECTR and SHERLOCK assays; **C** Sequence-specific target binding. Catalytically inactive Cas proteins bind to the target gene that is complementary to gRNA. **D** Sequence-specific target cleavage. Cas proteins cleave the target gene, followed by the sequence-specific binding. **E** Target-specific trans-cleavage. Some Cas proteins such as Cas12a or Cas13a non-specifically cleave the ssDNA or ssRNA nearby upon binding to the target gene. **F** Three widely-used signal detection techniques: the fluorescence, colorimetric or electrochemical signal can be monitored to detect the existence of the analytes (Figure modified after Kim et al., Biomolecules 2021, 11(8); Copyright: CC BY License)

The collateral cleavage of nucleic acids has opened a new chapter in sensing of diverse targets such as genetic elements, disease markers, pathogenic agents, and other biomolecules using nucleic acids as molecular recognition elements. For example, CRISPR-Cas12a-based DETECTR, HOLMES, and CRISPR-Cas13a-based SHERLOCK assays (Fig. 2) are designed for this purpose [5, 6]. The CRISPR-Cas tools are easy to design and construct, moreover, it possesses high specificity and sensitivity. Therefore, these assays could be incorporated into a portable format as point-of-care (POC) diagnostics tools.

Before 2019, CRISPR-Cas sensors could only recognize nucleic acid markers. An impediment was developing a system to identify and bind specifically to various non-nucleic acid targets not directly recognized by Cas proteins [7, 8]. To overcome this hurdle, a study demonstrated short ssDNA sequences (such as, a fragment of DNA aptamer); can serve as an “activator DNA (acDNA)” to initiate CRISPR-Cas12a trans-cleavage activity [9]. Use of acDNA molecules catapulted CRISPR-Cas applications for non-nucleic acid molecules by integrating aptamers as molecular recognition elements (Table 2).

SELEX is commonly used for aptamer discovery and produces highly specific aptamers against target molecules, where the aptamers are short fragments of nucleic acids (ssDNA or RNA) sequences that attach to their targets with a high binding affinity [10–12]. Aptamer possesses several merits over other molecular recognition elements, for example, ease of in vitro synthesis, amplification, sequencing, fluorescent labeling, chemical modifications, and modular design. The aptamers have applications in a wide range of fields. Over several hundred precise and characterized aptameric sequences are available for the detection of small molecules, proteins, live cells, pathogens, metal ions, pesticides, and antibiotics ([13–16]). Many aptameric sensors are available for screening in biomedical and life sciences and have been helpful for analytical chemistry, environmental, and food analysis [17]

Optimizing the CRISPR-Cas effectors for aptamer-based biosensing has opened new doors in molecular diagnostics. The inclusion of aptamers for high-affinity detection of more comprehensive targets enables direct measurement of a signal as a result of a binding event of an aptamer to the target molecules and relaying it in CRISPR-Cas supported signal enhancement by collateral cleavage of ssDNA probes. CRISPR-Cas-based diagnostics, aptamers facilitate sensing of non-nucleic acid targets. In general, an ssDNA plays the role of activators as crRNA could recognize aptamer; depending on target recognition or binding detection and quantification of oligonucleotides is possible; and collateral damage provides a direct readout from reporter probes. Here, we provide holistic coverage of advancements in aptamer-based CRISPR-Cas sensors. This review presents the basics of the CRISPR-Cas12 system and aptamer, including the necessary components of CRISPR-Cas for diagnostics (Fig. 2). Then, we focus on signal generation strategies using fluorescence modifications, colorimetric assays, electrochemical, nanomaterials (gold nanoparticles, nanosheets, magnetic particles, etc.), Rayleigh-and Raman scattering for diagnostics.

## Fundamental concepts of CRISPR-Cas-based biosensing

The polymorphic genes and Cas proteins, which form the basis of CRISPR-Cas technology, are characterized by the presence of palindromic sequences, protospacer motifs, and an upstream leader sequence in the promoter regions. With unique activity, Cas proteins, and the mechanism of CRISPR-Cas, it is classified as Class 1 and Class 2. Class 1 is a multi-factor effector system that necessitates several Cas protein subunits and is less amenable; however, the Class 2 effectors have a simple component and depend on a single Cas protein which forms the basis of diagnostics applications. For the design of the CRISPR-Cas diagnostics assay, a Cas protein, crRNA, an activator DNA, a labeled reporter, and the target specific ssDNA aptamers are required.

**Fig. 3:**
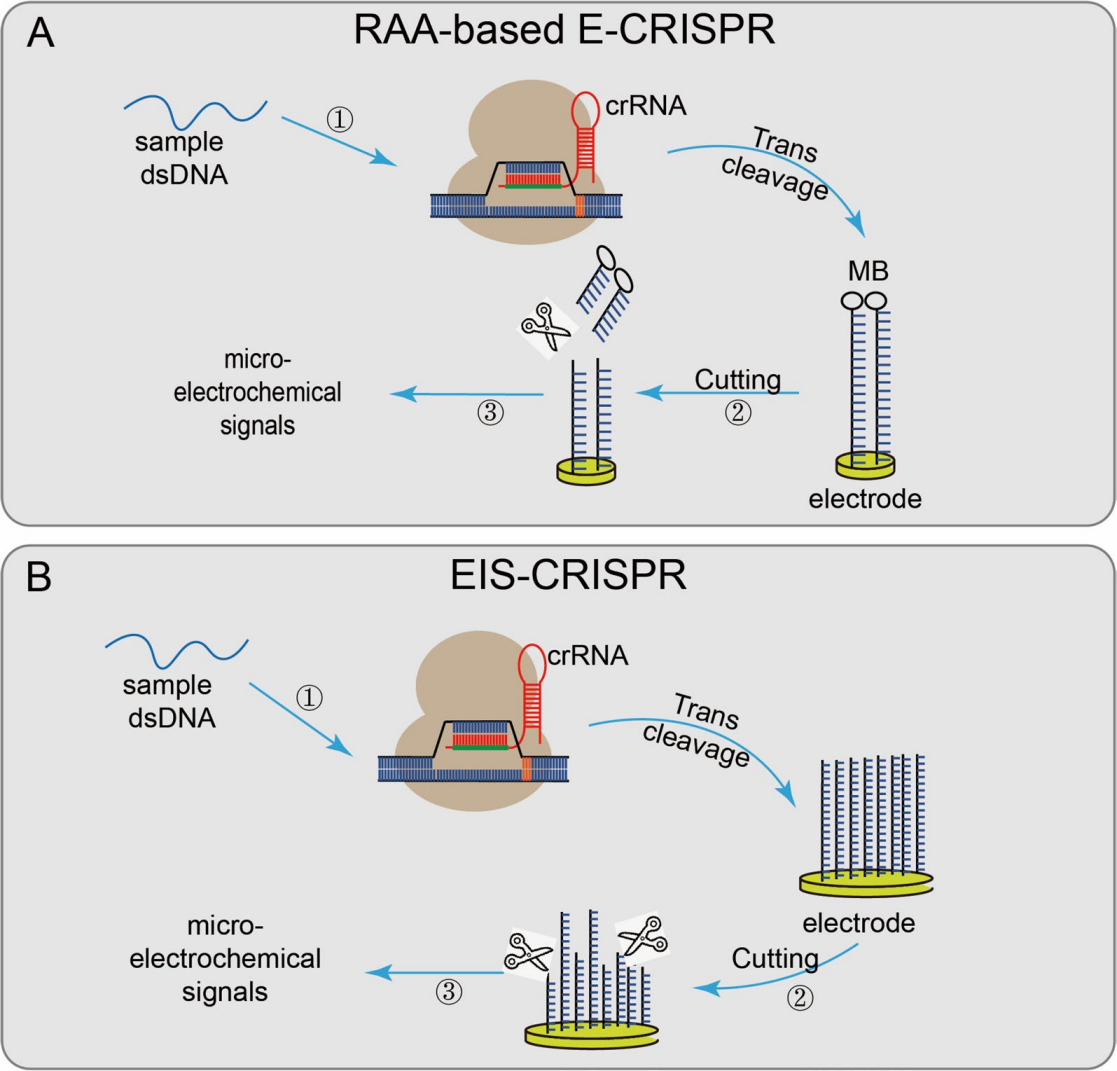
**Applications of CRISPR/Cas12. A**. RAA-based E-CRISPR, uses MB to modify the ssDNA reporter gene and assemble it on the working electrode, the sample is first amplified by RAA, when the target sequence exists, non-specifically cleaves the MB-modified reporter gene on the electrode surface, finally analyzed by SWV to measure the microelectrochemical signal before and after the introduction of the target nucleic acid sequence; **B**. EIS-CRISPR, fixes ssDNA on a gold electrode to limit the electronic communication between the electrode and the solution; when the target DNA exists, the Cas12/gRNA system binds to the target DNA and trans-cleaves the ssDNA on the gold electrode and accelerates the electron transfer between the electrode and the solution, detecting subtle changes in the electrode surface current at last (Modified after Li et al., Diagnostics 2022, 12(10); Copyright: CC BY License)

**Fig. 4:**
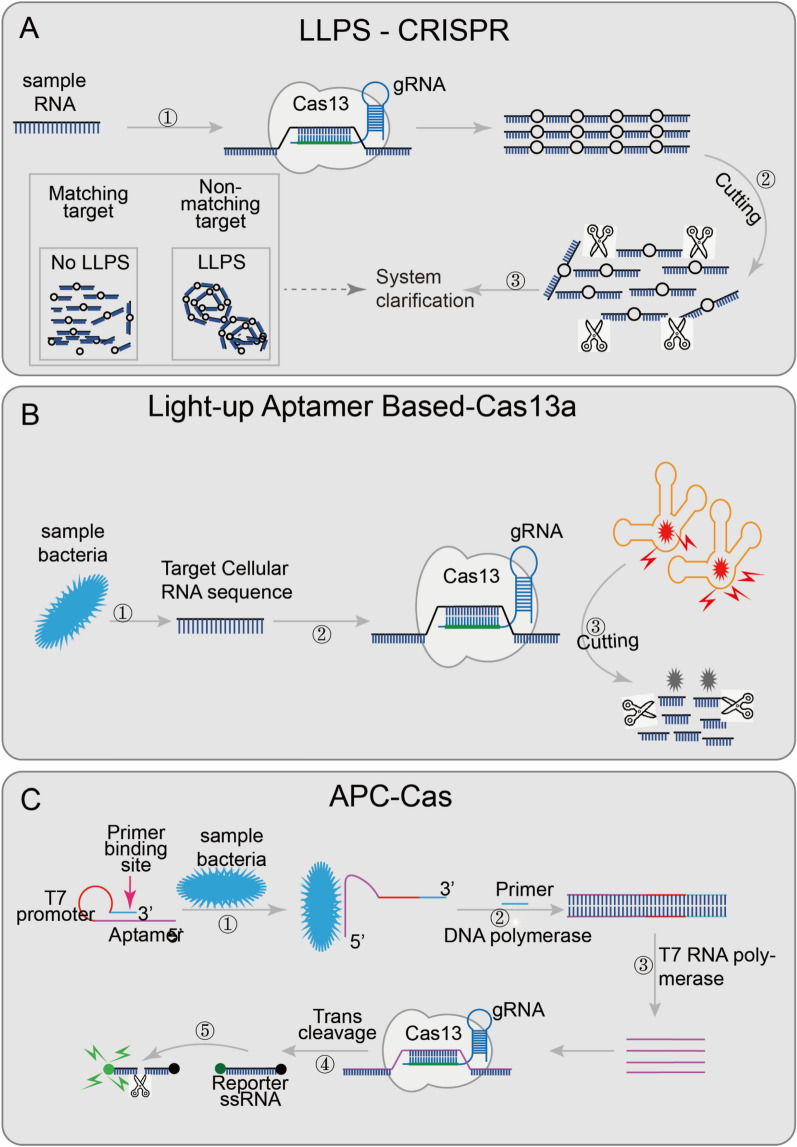
**Applications of CRISPR/Cas13 and CRISPR/Cas14 technology.** **A** LLPS-CRISPR, combined with the collateral cleavage activity of Cas12a/Cas13a, cleaves long-chain into short-chain nucleotides when the target sequence is present; then the solution will become clear afterwards; **B** Light-up aptamer-based-Cas13a introduces a new light-up RNA aptamer broccoli/DFHBI-1T complex; when the target sequence is present, Cas13a digests the aptamer broccoli, and the high-fluorescence bound-state DFHBI-1T becomes the low-fluorescence free state; **C** APC-Cas’s aptamer domain will specifically recognize and bind to the target pathogen, so that AP expands from a hairpin-like inactive structure and transforms into an active structure; the primer domain can be combined with the primer, and then, with the participation of DNA polymerase, AP is used as the template chain to generate dsDNA, which replaces the target pathogen and realizes the first amplification; then the T7 promoter domain is amplified by T7 RNA polymerase to achieve the second step of amplification; subsequently, the Cas13a/crRNA complex recognizes the ssRNA produced by the second step and non-specifically cleaves a large number of surrounding RNA gene reporter probes, achieving the third step of amplification, finally generating a fluorescent signal (Figure modified after Li et al., Diagnostics 2022, 12(10); Copyright: CC BY License)

**Fig. 5:**
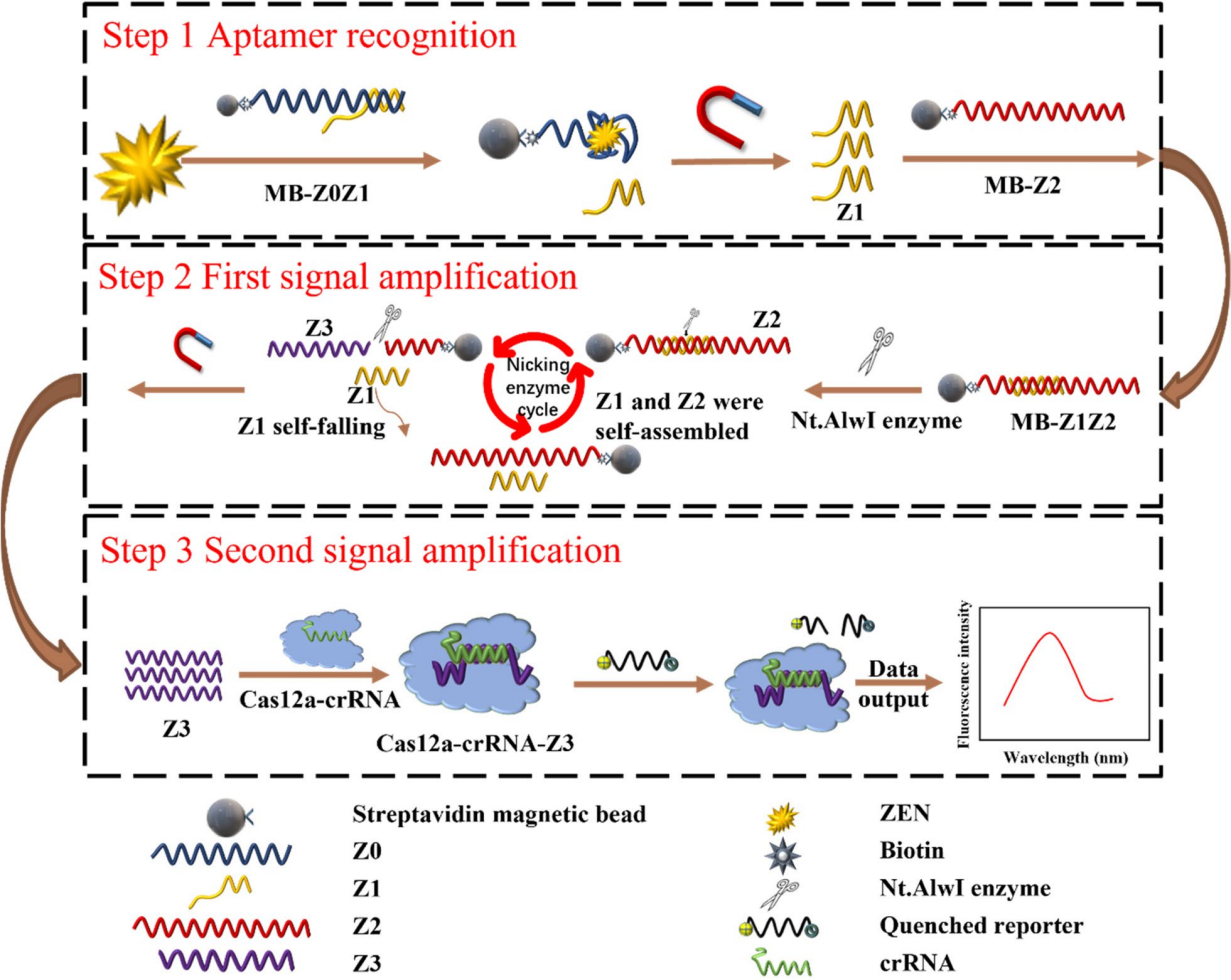
. **Magnetic-bead-assisted dual-signal-amplification aptasensor for sensitive ZEN detection based on the Nt.AlwI enzyme and the Cas12a enzyme.** Step 1: The aptamer probe recognizes the ZEN toxin and causes Z1 to dissociate into solution by competitive binding. Step 2: After Z1 and Z2 were hybridized, the cutting activity of the Nt.AlwI enzyme was activated, the Z2 chain was cut to release Z3, Z1 was self-shed after the cutting was finished and it hybridized with Z2 again, and a large amount of Z3 was released by the enzyme-cutting signal amplification to achieve the first signal amplification. Step 3: The combination of Z3 and the Cas12a-crRNA complex activates trans-cleavage activity, non-specifically cleaving any ssDNA so that the added fluorescent signal molecule was cleaved and the quenched fluorescence was restored (Figure from Yao et al., Foods 2022, 11(3); Copyright: CC BY License)

The examples of Class 2 Cas proteins include Cas3, Cas9, Cas10, Cas12a (Fig. 3), Cas13a (Fig. 4), and Cas14a (Fig. 5). Among these, Cas12a is most commonly used in biosensing, it is a single guide RNA-mediated DNA nuclease with two unique domains: a Nuc and a RuvC [2, 18] RuvC domain is involved in target recognition and facilitates the cleavage activity by Nuc lobes. Cas12a can be activated either by dsDNA or ssDNA and can degrade both the specific target sequence and the nonspecifically (collateral damage) any sequence. A protospacer motif region (PAM) is essential for binding to dsDNA targets, while a PAM sequence is not required for ssDNA. Among several Cas proteins, Cas12a is commonly used in aptamer-based sensing. Another Cas protein, Cas13a, recognizes RNA as a target and requires a single RNA; it also possesses two separate domains for target recognition and RNA degradation. Cas13a enzyme digests flanking RNA sequence next to crRNA on complimentary site and also cleaves ssRNA in a nonspecific manner; commonly employed for viral analysis. Another Cas protein, Cas14a, is highly compact and much smaller than Cas9; can target and degrade ssDNAs nonspecifically without need of a target sequence. Moreover, Cas14a has shown a high affinity towards ssDNAs than Cas12a and could degrade long ssDNA probes. Cas14a is a newly found enzyme used to analyze various targets [19, 20].

**Fig. 6:**
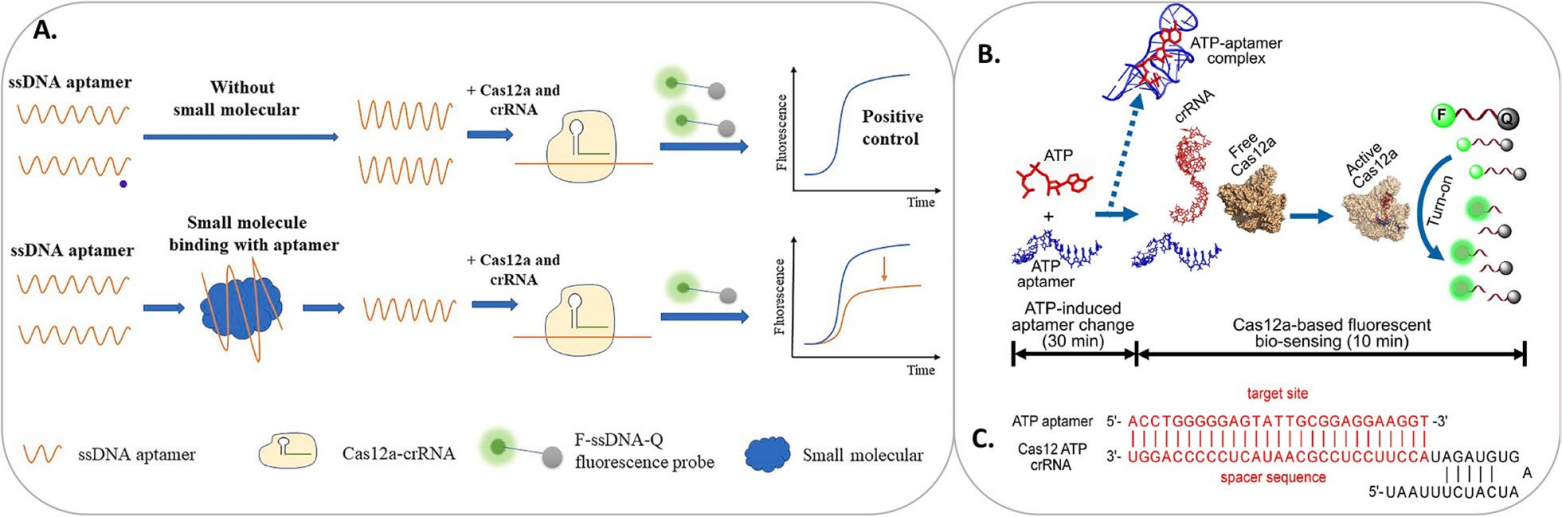
**The small molecule diagnostics.** A Generalized schematic of the molecular radar strategy for small molecules diagnostics (Figure from Niu et al., Biosensors and Bioelectronics 183 (2021) 113196; Copyright by Elsevier, used with permission). **B **Proposed CRISPR-Cas12a biosensor for ATP detection; **C** The schematic of target ssDNA as well as crRNA used; the target site is highlighted in red (Figure from Peng et al., Sensors & Actuators: B. Chemical 320 (2020) 1281642; Copyright by Elsevier, used with permission)

The major limiting factor in the CRISPR-Cas system is the design of effective crRNA, which facilitates target recognition, binding, and cleavage efficiency [21]. The crRNA nucleotide composition, sequence, and length need careful evaluation for a successful outcome of the diagnostic assay. The crRNA contains two functional domains of a guide region and an activator sequence. Cas12a, the guide region sequence which forms the scaffold is 5’-UAAUUUCUACUAAGUGUAGAU-3’ (Fig. 6). The guide sequence, which forms the basis of crRNA and makes a binding scaffold, helps the Cas enzyme and varies according to the Cas protein. In Cas13a, this segment carries a sequence of 5’-ACCCCAAAAAUGAAGGGGACUAAAA-3’. An ssDNA activator sequence is used for Cas proteins, usually designed with complementary a fragment of target nucleic acids such as aptamer sequences. The molecular identification and efficient binding to the activator is a prerequisite to proceed collateral cleavage of fluorophore-modified reporter DNAs. The reporter modifications vary from fluorophore-quencher (F:Q) pair to nanoparticles to antibodies or affinity tags at 5’ or 3’-ends (or both terminals). Additionally, the molar ratio of the Cas protein to crRNA has to be carefully adjusted for efficient signal amplification [2, 5, 8].

**Fig. 7:**
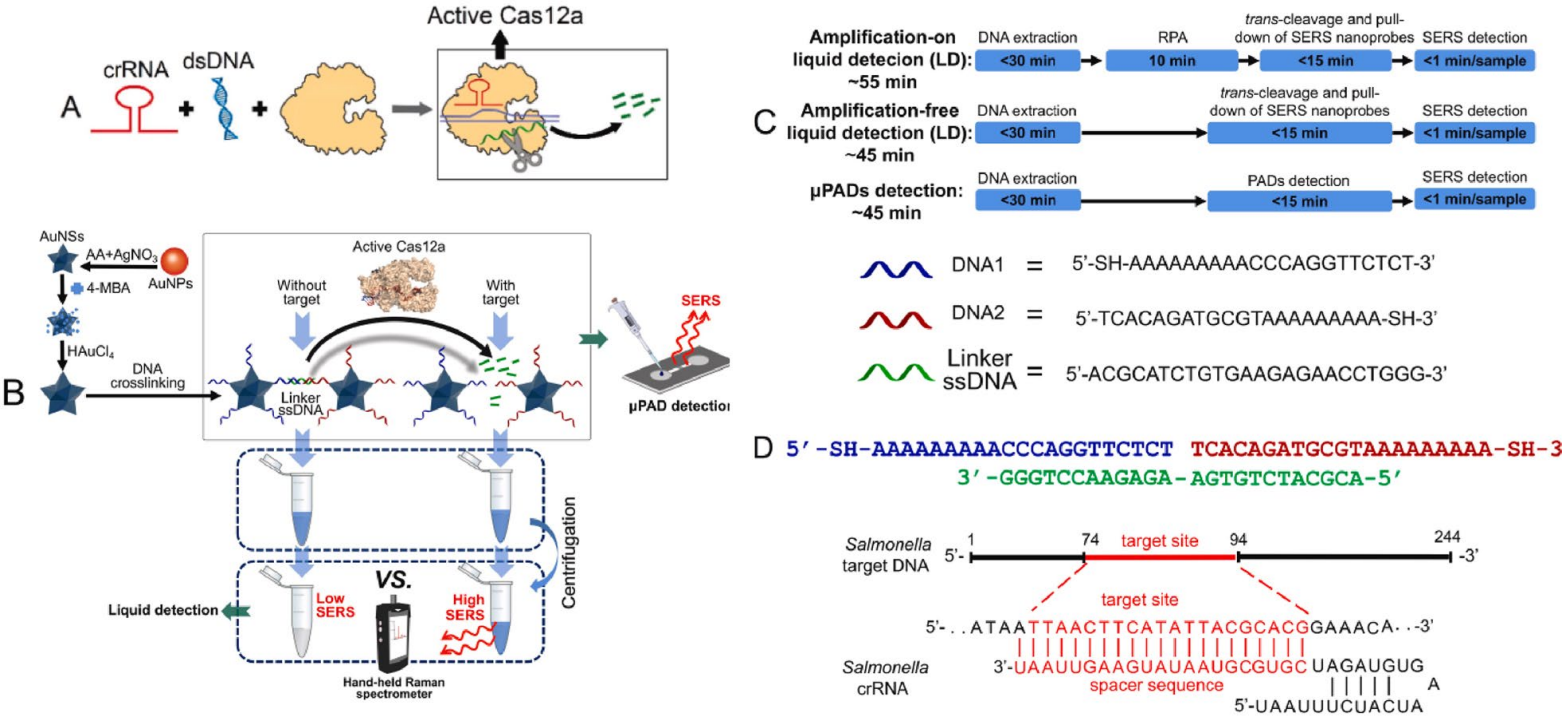
**The principle of Raman spectrometer-read CRISPR/Cas biosensor for nucleic acids detection of pathogenic bacteria.** **A** The activation of CRISPR/Cas12a for trans-cleavage. The green ribbon represents single-stranded DNA subject to trans-cleavage. **B** The preparation of gold nanostar@4-mercaptobenzoic acid@goldnanoshell structures (AuNS@4-MBA@Au) and their utility in combination with CRISPR/Cas12a for SERS-based bacterial detection for both in-tube and μPAD detection. DNA1 and DNA2 were colored as blue and red, respectively and linker ssDNA was green. **C** The schematics of the biosensing processes with the estimated assay time for each step. **D** The nucleic acid sequences required for the proposed biosensor and the hybridization of linker ssDNA with DNA1 and DNA2.* AA*  ascorbic acid. (Figure from Zhuang et al., Biosensors and Bioelectronics 207 (2022) 114167; Copyright by Elsevier;used with permission)

The aptamer-based sensing of the target has reached its saturation; hence, there is a necessity for signal enhancement and diversification of diagnostics for field-level testing. The past couple of years has seen a rise in applications of CRISPR-Cas proteins biosensing due to rapid and specific detection potential [8]. Moreover, the combination of CRISPR-Cas with aptamers provides solutions because they are quick, simple, accurate, modular, dynamic, and cheap. Additionally, these sensors can be used in compact assembly with portable biosensing [22, 23]. Several signal generation and transduction are demonstrated by coupling with aptameric sensors, including use of novel nanomaterials for electrochemical, fluorescent, colorimetric, and SERS sensors (Fig. 7). In following section, we discuss some of the most commonly used signal detection approaches.

## Fluorescence-based sensing

The development of fluorophore-modified aptameric sensors brings agility and ease of conducting assay due to increased sensitivity and availability of wide range of signal detection devices. Fluorescence analysis is one of the key technique in molecular diagnostics. Several strategies are found to construct aptamer-based CRISPR-Cas fluorescent sensors, for instance, direct detection, sandwich design, and allosteric hairpin (AH) mediated detection. The direct sensing strategy depends on Cas enzymes’ potential to damage collaterally via binding with ssDNA activator regions to crRNA; there is no need for pre-amplification steps. Two ways direct binding and detection can be performed using aptamers: direct-activation strategy and locked-activated strategy. This detection strategy uses a short activator ssDNA (acDNA) sequence to facilitate CRISPR-Cas binding. The reporter sequences are dual labeled with a fluorophore and a quencher at both ends, and start with the quenched fluorescence. One of the most commonly used F:Q pairs is Fluorescein-Black Hole Quencher 1 (FAM-BHQ1). Upon binding of activator DNA to the ribonucleoprotein complex formed by Cas12a-crRNA, the activation of Cas enzyme takes place, and the collateral cleavage of the F:Q reporter by Cas12a begins, which in turn produces intense fluorescence. The fluorescence signal is measured and quantified (or could be used for presence and absence in visual analysis). As aptamers are specific to the target molecules, in the presence of the target, they would form a high affinity binding complex with the target, and the acDNA would be released, resulting in concentration-dependent cleavage by Cas12a. The assay could be used in the opposite manner, where signal yield is directly proportional to free-aptamer concentration; the approach has been devised for ATP detection [24]. Some factors affecting CRISPR-Cas detection were identified, including the concentration of Mg^2+^ ions and the ratio of acDNA. While developing CRISPR-Cas for sensing Mg^2+^ ions using aptamers, the effect of ionic strength was noticed, which was found to play a role in the conformation of the RuvC domain [25].

Nonspecific or background signals present unnecessary hurdles in fluorescence analysis using aptamer-based CRISPR-Cas detection. To overcome this challenge, a locked-activated approach was designed in which a complementary strand of aptamer acts as an acDNA. In this design, the structure-switching approach of ssDNA aptamer is exploited, where a complementary acDNA probe is allowed to hybridize with the aptamer [26]. In the presence of the target, the aptamer preferentially binds to the target molecule, and that would release acDNA. By direct strategy, the acDNA binds to CRISPR-Cas and activates the nuclease activity. The approach could differentiate live vs. killed dead bacterial cells using aptamer-Cas14-a1 [19, 20].

For successful detection based on acDNA, optimal probe design is essential. Using partial base pairing in ATP aptamers (Fig. 6B, C) to lock acDNA with a sandwich probe of a1-acDNA-a2 (aptamer1-acDNA-aptamer2) [19]. Similarly, an excellent onsite aptasensor toolkit was developed that displayed high sensitivity of 38 nM to melamine, compared to single acDNA activation approach [27]. Thus, the sandwich probe in technique was proven to be better for increased sensitivity. In another study, dsDNA as the acDNA elevated the collateral cleavage ability of Cas12a than ssDNA to a higher level [18].

Antibody-based enzyme-linked immunosorbent assay (ELISA) is a popular analytical approach [28]. Modified ELISA has used aptamer as an alternative to antibodies (ELASA) [29]. Aptamers are easier to load onto a plate and label with a variety of reporters, linkers, and functional groups, making signal transformation more efficient than an antibody. CRISPR-Cas coupled with ELASA, now called CLASA, provides even more sensitive and practical applications [29, 30].

Three types of sandwich design strategies are employed in ELISA—antibody-target-antibody (anti-T-anti), anti-T-aptamer (anti-T-apt), and apt-T-apt. The indiscriminate cleavage activity of the Cas enzyme can overcome HRP’s detection limit in ELISA. For example, using an anti-T-anti sandwich biosensor and antibody-dsDNA as the acDNA for human IL-6 and VEGF, a highly accurate detection with more than 100 times powerful compared to ELISA was achieved [31]. Similarly, Li et al. [29] adopted the apt1-T-apt2 sandwich strategy to improve upon this technology. In some cases, when targets have multiple aptamers, the “apt1-T-apt2” strategy becomes obsolete. Therefore, an “anti-T-apt” sandwich was proposed in combination with Cas enzymes [32]. Most significantly, optical fiber instead of PS was used to form a sandwich of fiber/anti-T-apt/ Cas-crRNA, which was able to detect interferons with over 1000-fold higher sensitivity compared to ELISA [33]. To combine the Cas sensitivity with PCR technology, in situ PCR amplification after sandwich formation to increase acDNA and CD109 aptamers served as templates [29]. The PCR dsDNA product and a crRNA activated the downstream Cas12a system.

## Electrochemical-based sensing

Being highly sensitive, easy to handle, cheap, modular assembly, portability, and rapid signal detection, the electrochemical sensors have captivated researchers’ attention and made waves in CRISPR-Cas-based analysis for aptamer [34]. For electrochemical sensing, direct target recognition, label-free analysis, pre-amplification free, and availability of novel electrode materials make it a lucrative option for integration in aptamer coupled with Cas sensing. For example, Dai et al. [9] created a Cas12a-based EC sensor with aptamer as the acDNA, captured by crRNA to start collateral cleavage. In which methylene blue was attached to one end for electrical signal transduction, while a thiol moiety helped to link another end on the electrode. Cas12 cleaved off the methylene blue (redox probe) and detached from the electrode surface, reducing the signal; using this strategy, TGF-b1 protein was detected with a sensitivity of 0.2 nM [35]. Additionally, an electrochemiluminescence (ECL) sensor using Cas and aptamer sensing was designed [36]. Electrochemical sensing usually needs electroactive labels and a sensitive interface. Abnous et al. created a label-free aptamer-based CRISPR-Cas supported EC sensor by employing acDNA with TdT [37], which allowed the redox probe of [Fe(CN)6]^3−/4−^ to react with the surface, producing a quantifiable signal of cocaine binding to the aptamer. Using a similar approach, Liu et al. [38] designed EC impedance spectroscopy with Cas12a substrate and in situ RCA amplification on a gold electrode to detect nucleocapsid protein at picogram per mL concentrations.

A preassembled EC module was used to increase signal [38, 39], where the HCR (hybridization chain reaction) product peripheral is exposed to a lot of acDNA to promote collateral cleavage activity. A modified approach of immuno-RCA assembly multiplies signals from long ssDNA for bacterial strain-specific aptamers and targets repetitive acDNAs. Further, a sandwich-type “apt1-T-apt2” CRISPR sensing on AuNPs@Ti_3_C_2_T_x_-Mxene surface and aptamer for VEGF could detect sub-picomolar range [31]. To overcome some of the limitations of these assays, an immobilization-free EC sensor with stacking interaction between DNA molecules and the reduced GO/GCE was established [40] and demonstrated for successful detection of thrombin with as low as single femtomoles. Large particle size modifications detach the substrate from the electrode, which hinders electron conduction and performance. Ultra-thin two-dimensional covalent organic framework nanosheets may have superior application in modifications due to their shorter charge transfer durations and distances, high exposure to surfaces, and active binding sites. To make use of aptamers and CRISPR-Cas effectors with HDA probe-triggered single-circle amplification, the detection of PD-L1 in exosomes at 38 particles per mL was recorded [41].

## Nanotechnology-based  sensing 

There are several nanotechnological strategies evolved for biomolecular detection. For example, use of gold nanoparticles (AuNPs) in the biological analysis is well known [42–47]. Zhao et al. designed an AuNPs-based nanoprobe for Cas sensing to improve acDNA carrier to gain fluorescence yield [48]. A sandwich structure of anti-T-aptamer/AuNP/acDNA was created that activated the trans-cleavage system. Higher loading on the AuNP surface leads to three times more sensitivity than free acDNA wither better accuracy. Li et al. assembled apt-acDNA as in hybrid DNA architecture (HDA) structure, with partial ssDNA sequences [29], carrying PAM sequence specific to promote cis-cleavage by Cas12a with the potential of 1000 times sensitivity over traditional Cas enzyme.

**Fig. 8:**
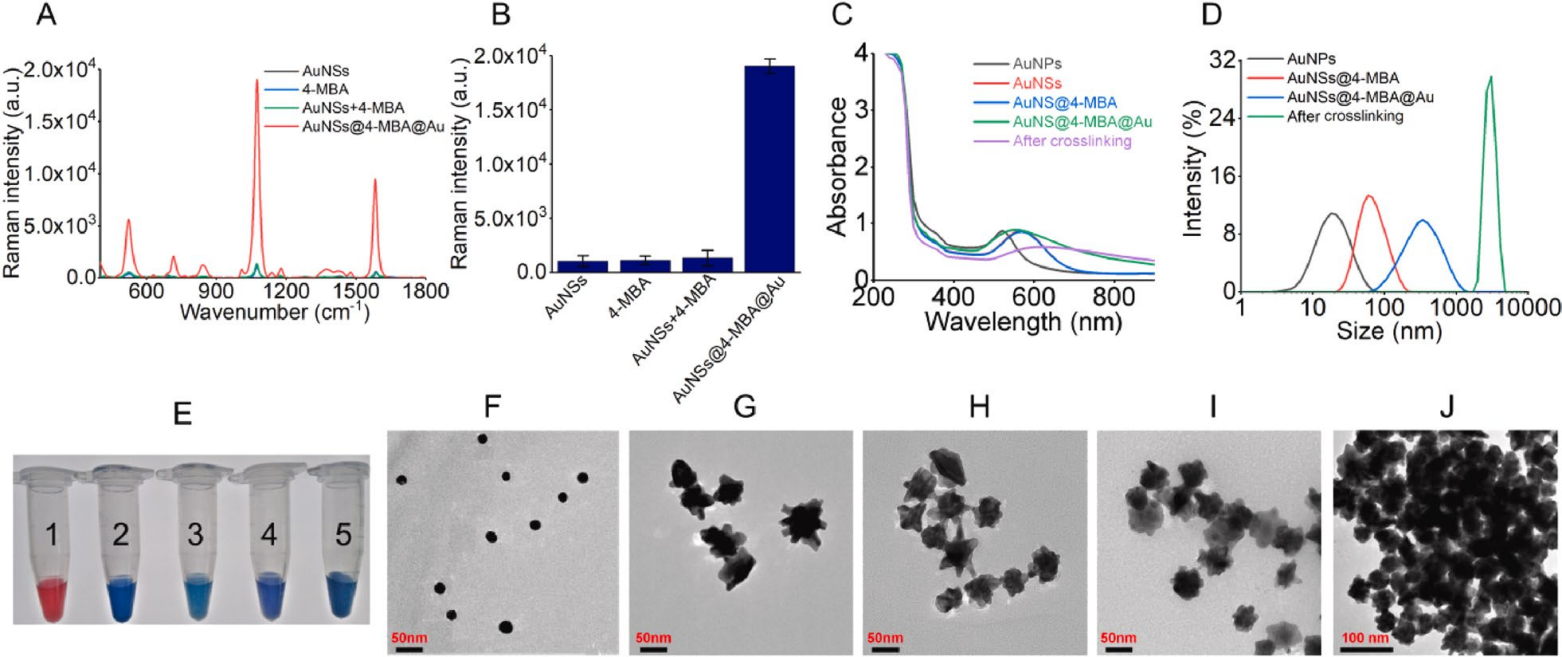
**The characterization of AuNS@4-MBA@Au and AuNS@4-MBA@Au@DNA (thiolated ssDNA conjugates). **Raman spectra (**A**) and histogram of SERS signals at wavenumber of 1075 cm-1 (**B**) for AuNSs, 4-MBA, physically mixed solution of AuNSs together with 4-MBA and AuNS@4-MBA@Au. **C** UV–Vis absorbance spectrum of each sample. **D** DLS profile of each sample. **E** Picture of each sample. 1: AuNPs; 2: AuNSs; 3: AuNS@4-MBA; 4: AuNS@4-MBA@Au; 5: AuNS@4-MBA@Au@DNA. TEM images of AuNPs (**F**), AuNSs (**G**), AuNSs@4-MBA (**H**), AuNS@4-MBA@Au (**I**) and the crosslinked AuNS@4-MBA@Au@DNA (**J)** (Figure from Zhuang et al., Biosensors and Bioelectronics 207 (2022) 114167, Copyright by Elsevier; used with permission)

In addition to AuNPs, magnetic nanoparticles, such as magnetic beads (MB) (Figs. 7 and 8) are popularly used in diagnostics [49, 50]. The use of MB for capture or carrier and enrichment in aptamer sensing is highly beneficial. While combining aptamers with CRISPR-Cas, MB can be used to convert signals, separate, or reject non-target molecules such as DNA or RNA. MB with a high surface-to-volume ratio can potentially increase acDNA transport [49].

Linking of ssDNA aptamer to MBs via streptavidin (SA)-biotin binding was found to outbid MB-HDA dissociation [50]. Upon magnetic sorting, the conjugates retained free-complementary strands and retained acDNA collateral cleavage activity. Such magnetic sorting made sure acDNA is capable of catalysis without off-target or unexpected cleavage by inappropriate DNA hybridized structure formation with crRNA. The MB nanoparticle-assisted method has demonstrated great promise for several targets, such as microcystin-LR detection, toxic lead ion detection, and miRNAs analysis [51]. Furthermore, using a modification of DNA hybridization to MB and Cas enzymes, several aptamers were employed to detect variable targets such as cocaine, alpha-fetoprotein, and SARS-CoV-2 viral particles [52, 53].

Connecting a target to higher CRISPR-Cas activators (ssDNA or dsDNA) improves sensitivity, towards this rolling circle amplification (RCA) was employed [54], where SA/MB/Apt-A captured protein A-positive bacteria by magnetic separation, and then target-specific methicillin-resistant staphylococcus aureus (MRSA) were identified by enrichment of the penicillin-binding proteins 2a (PBP2a) with apt-B. In turn, complementary DNA was released and involved in cyclized padlock by hybridizing with its two terminals and triggering the following RCA assisted by T4 DNA ligase. Moreover, the strategy was exploited using Nt.AlwI endonuclease to obtain multiple copies of acDNA, which improved the sensitivity of ZEN toxin [55]. Similarly, Wang et al. used hydrazone ligation in a three-dimensional DNAzyme walking nanomachine to generate more acDNAs to amplify trans-cleavage activity [56]. It is a versatile tool for understanding molecular behavior and mobility. Its high nanoparticle surface-to-volume ratio enabled signal enhancement and freely available acDNA boosted downstream collateral damage after magnetic separation that could detect lipopolysaccharide with 7.31 fg/mL detection limit [57].

Recently, an MB-multivalent duplexed aptamer module has been shown to detect PTK7, a cancer biomarker using Cas enzyme. Using rolling circle amplification (RCA) and preassembled target-specific aptamer on the surface of MB to elongate ssDNA strands; resulted in very high collateral damage activity. Similarly, to overcome the slow release of acDNA, an assay performed using hybrid DNA for exponential signal improvement; repeated acDNAs enhanced frequency and accessibility to Cas12a/crRNA complex and increased sensitivity [58]. Using this approach, SARS-CoV-2 RNA was detected to be as low as ~ 42 copies/mL. To simplify the multi-step process as described earlier, a wash-free homogeneous allosteric hairpin probe (using single, dual, and ternary) circle amplification was proposed. Using single-circle amplification [59, 60], an AH probe mediates strand displacement amplification with aptamer, nicking enzyme cutting site, and signal transduction. The aptamer could find the target and unzip the AH probe, revealing two regions to allow the formation of a primer junction. Employing KF polymerase catalysis, dsDNA was generated and could be recognized by Nt. BbvCI to be digested as an acDNA fragment, further amplified collateral damage. This permitted detection of tobramycin with high sensitivity up to picomolar range. Enzyme-free dynamic DNA network catalysis was used in another study to multiply acDNA copies [61], bypassing the complicated polymerase/enzymatic reaction. The inclusion of T7 RNA polymerase and CRISPR-Cas13a triggered the reaction, as demonstrated in aptamer application for bacterial detection of 1 CFU, a level 40 times better than RT-PCR [24, 62]. Similarly, dual and ternary circle-based the CRISPR-Cas sensor detected various targets raging from extracellular vesicles trace level of ATP [24].

The sequential mixing reduced the number of preparatory steps and increased reproducibility. 2D nanomaterials, such as metal carbide (MXene) nanosheets with high surface area, act as efficient quenchers [63] and minimize background signals. Sheng et al. designed a flexible PAM domain with dsDNA probes as the acDNA achieved super-quenching to quantify picograms of lipopolysaccharide and two-digit Gram-negative bacteria. Further, 2D nanosheet and Cas14a coupled to aptamer and porphyrin metal–organic framework nanosheets as the quencher was able to detect MC-LR at very low levels [64–66].

## Colorimetry-based sensing

The fluorescence and EC assays are dependent on electronic devices and expensive designs. A signal readout that the naked eye can visualize makes appealing alternatives for resource-limited point-of-care settings [46, 47]. Several colorimetric assays with DNAzyme-based colorimetry, nanoparticle aggregation, and colorimetric strips are being developed [45, 46].

Integration of optical and visual detection into CRISPR-Cas12a using an HRP-mimicking DNAzyme that formed the sandwich complex of PS/apt1-T-apt2/acDNA and activated the cascade reaction of hemin-peroxide, tetramethyl benzidine (TMB) [67] for visualizable color change produced sensor with 1.5 X 10^6^ times sensitivity for ATP detection [24]. Moreover, this approach was used in a sandwich design of PS/antibody-T-apt/acDNA to detect several targets such as CEA protein, bacteria, and norovirus [68]. Additionally, due to the peroxidase-mimic activity and distance-dependent optical behavior of AuNPs, they have been found in use in the construction of colorimetric sensing. For example, AuNPs coupled with Cas12a collateral digestion and RCA amplification were used for colorimetric CRISPR-Cas sensing, where aptamer/crRNA/Cas12a ternary complexes cleave primer sequences and padlock probes modified on AuNPs. Wang et al. [69] used distance-dependent optical properties of AuNPs and nicking enzyme-free amplification to produce more acDNA and detected aflatoxin M1 (AFM1) with ppb level of accuracy and sensing of serum PSA [69, 70].

Lateral flow assays (LFA) or paper-strip designs based on CRISPR-Cas effectors can cleave products and incorporate AuNPs for colorimetric readout signals. For instance, an MC-LR strip using FAM and biotin dual-modified ssDNA as the intermediate reporter was developed [65]. The target caused the cascade reaction and Cas12a trans-cleavage, resulting in FAM- and biotin-ssDNA segments. The reporter and cleaved FAM-ssDNA were conjugated to anti-FAM-coated AuNPs as they migrated along the strip. Additionally, the common pregnancy strip tests (PST) targeted at the detection of human chorionic gonadotropin (hCG) [ 22, 71, 72], have found different usage. Like, Tang et al. [73] developed a novel NHP probe that could hybridize with cauliflower-like large-sized DNA assemblies (CLD). The target-induced cleavage event prevented the complex CLD-NHP from forming, and the cleaved NHP probe migrated on PST with a red T line. This clever design detected adenosine in colorimetric fashion using the naked eyes.

## Other sensing approaches

In addition to the above approaches, several CRISPR-Cas target sensing mechanisms are integrated with ssDNA aptamer to generate unique signal transduction modules that provide precise and reliable analytical alternatives. To name a few methods include luminescence resonance energy transfer (LRET), light-up RNA, resonance Rayleigh scattering (RRS), and surface-enhanced Raman scattering (SERS).

Luminescence resonance energy transfer (LRET) based sensing can overcome background interference, offering a strong enough capability to handle complicated biological samples [74]. Lin et al. used ssDNA-UCNPs as reporters and gold nanoparticle-modified Ti_3_C_2_T_x_ MXene-AuNP nanosheets as quenchers to create an LRET adsorptive quenching sensor [75]. in the absence of target, the acDNA interact and initiate Cas12a to perform collateral digestion of ssDNA conjugated to upconverted nanoparticles (UCNPs), and get adsorbed on MXene-AuNPs that would retain upconverted of luminescence (UCL). While Cas12a action is blocked if the target is present, non-cleaved reporters bind to MXene-AuNP, resulting in a quenching effect. Deoxy-nivalenol was detected at 0.64 ng/mL by the sensor; the method achieved ultra-sensitive detection of ATP [68] and cardiac troponin I (cTnI) [76] For ochratoxin A (OTA) detection, Mao et al. developed a UCNP-MB probe [77] making feasible for OTA bound with aptamer and unfolded HDA probes to release complementary DNA and initiate trans-cleavage action. After magnetic separation, OTA was detected with the sub-ppb level of sensitivity in CRISPR supported assay.

Introducing RNA reporter probes like Broccoli that could bind DFHBI-1 T dye and switch on its fluorescence [78] with Cas13a by careful designing the crRNA provides [79, 80] the light-up RNA aptamer-based CRISPR sensor. It has the potential to replace expensive chemical modification and extensive synthesis steps with better quantification potential. Cas13a-catalyzed products cannot interact with DFHBI-1 T dyes, resulting in a “turn-off” signal. The light-up RNA sensor could detect bacteria and was useful for the differentiation of living vs. dead bacterial cells with very low CFUs.

Gao et al. introduced a G-wire assisted non-cross-linking HCR reaction to create a label-free resonance Rayleigh scattering (RRS) CRISPR-effector powered aptameric sensor system that could reveal the molecular size, shape, conformation, and interfacial features [81]. When the target was present, the aptamer containing the PAM segment specifically recognized the target rather than crRNA/Cas12a system, suppressing trans-cleavage activity and triggering the HCR reaction by automatically aggregating reporter probes. The hyperbranched product produced signal amplification and very high RRS intensity. This approach detected LPS with accuracy and high specificity. Recently, Li et al. created a dual signal detection aptamer-based CRISPR-enzymatic paper strip for colorimetric and Raman scattering-based diagnosis; for which biotin-ssDNA-digoxin as the intermediate reporter was used and digoxin antibody-SERS tags (Au@BDT@Au) were exploited for generation of readout signals [82–84]. Such Raman sensing strips are easy for batch production, long-term stability, short sample demand, and cost-effectiveness; however, the unavailability of hand-held Raman devices may limit the diffusion of this innovative technology.

## Future directions and conclusions

The high affinity and specific binding properties of aptamers along with versatile CRISPR-Cas effectors make it idealistic sensors for detection and quantification. CRISPR-Cas enzymes are poised to impact and advance aptamer-based detection of various biomarkers and small toxic compounds. Although both technologies have seen exponential growth in a proof-of-concept, it would be interesting to see how field-level applications evolve in the coming future. The sandwich-type CLASA using nanoprobes for signal enhancement and preassembly for amplification-free detection will continue to develop in the coming years. Colorimetric strips, portable designs, and smartphone-based optical sensors are desired to simplify detection in POC settings. The aptamer-based CRISPR-Cas platforms have been demonstrated for analysis in biomedical sciences, environmental monitoring, food safety, and clinical diagnosis. Although several biosensors have adopted CRISPR-Cas effectors and exhibited their performance, critical technical issues still need to be addressed. For example, Cas effector proteins use different sequences to detect targets, so their crRNAs have changed activated regions, requiring complex optimization with activator sequences to initiate Cas enzymatic activity efficiently. CRISPR-Cas effectors' cleavage potential is also affected by the ambient environment, pH, buffer, salt concentrations, etc. Hence, optimization of assay conditions offers a challenging task for POC setting analysis. The design of crRNA, cost and time for producing Cas effectors, concentration of crRNA, and the ratio with reporters need to be minimized. These factors will determine the real-time and onsite detection affordably. Moreover, a multi-step process is involved in CRISPR-Cas and aptamer sensors; this must be streamlined to obtain robust readouts or signals. Therefore, the use of nanotools for colorimetric detection, LFA strips, and microfluidic chips provide attractive options to democratize the novel CRISPR-Cas and aptamer-based sensing for practical purposes. Overall, the advances in material science, molecular biology, enzyme engineering, and bioanalytical chemistry would help to design an ideal platform for next-generation diagnostics.

**Table 1:** Salient features of various Cas proteins used in diagnostics

Cas Protein	Class	Target	PAM	Collateral Activity	Refs.
Cas9	Class 2	dsDNA	NGG	No	[85]
Cas12a	Class 2	Both (ss/dsDNA)	TTTN	Yes (ssDNA)	[5, 18, 86]
Cas12b	Class 2	Both (ss/dsDNA)	TTN	Yes (ssDNA)	[6]
Cas13a	Class 2	ssRNA	–	Yes (ssRNA)	[5]
Cas13d	Class 2	ssRNA	–	Yes (ssRNA)	[87]
Cas14a	Class 2	ssDNA	–	Yes (ssDNA)	[3]

**Table 2:** Key representative examples of CRISPR-Cas proteins and aptamers in diagnostic assays of variety of targets

Target	Signal	CRISPR-Cas Effector	LOD	Refs.
DNA methylation	Fluorescence	Cas12b	10^–8^ nM	[6]
Extracellular vesicle	Fluorescence	Cas12a	100 particles/mL	[88]
Extracellular vesicles	Fluorescence	Cas12a	100 particles/µL	[89]
ATP	Fluorescence	Cas12a	0.39 μM	[67]
Na^+^	Fluorescence	Cas12a	0.21 μM	[67]
Aflatoxin B1 (AFB1)	Biolayer interferometry (BLI)	Cas12a	0.8 ng mL − 1	[90]
*Salmonella typhimurium*	Electrochemical	Cas12a	20 CFU/mL	[38]
Bacillus cereus	Fluorescence/RNA Light-Up	Cas13a	10 CFU	[91]
PDGF-BB	Fluorescence	Cas12a	0.75 pM	[29]
Telomere	Fluorescence	Cas9	–	[92]
17β-estradiol	Raman sensing/LFA	Cas12a	10 pM	[93]
Thrombin	Electrochemical	Cas12a	1.26 fM	[40]
ATP and Na^+^	LRET	Cas12a	~ 18 nM and ~ 0.37 μM	[68]
Prostate-specific antigen (PSA)	Colorimetric/AuNPs	Cas12a	0.030 ng/ mL	[69]
Cardiac troponin I (cTnI)	Fluorescence	Cas13d	12.6 pM	[87]

## Data Availability

This article presents the data generated and analyzed in figures or tables. Additional information on methods or materials used in this study will be made available upon request to the corresponding author.
